# Proper Balance of Small GTPase *rab10* Is Critical for PGC Migration in Zebrafish

**DOI:** 10.3390/ijms222111962

**Published:** 2021-11-04

**Authors:** Chengyu Mo, Wenjing Li, Kuntong Jia, Wei Liu, Meisheng Yi

**Affiliations:** 1 School of Marine Sciences, Sun Yat-sen University, Zhuhai 519082, China; mochy5@mail2.sysu.edu.cn (C.M.); liwj77@mail2.sysu.edu.cn (W.L.); jiakt3@mail.sysu.edu.cn (K.J.); 2 Southern Marine Science and Engineering Guangdong Laboratory, Zhuhai 519082, China; 3 Guangdong Provincial Key Laboratory of Marine Resources and Coastal Engineering, Guangzhou 510275, China

**Keywords:** primordial germ cells, *miR-202-5p*, *rab10*, migration

## Abstract

MicroRNAs (miRNAs) play important roles in post-transcriptional repression in nearly every biological process including germ cell development. Previously, we have identified a zebrafish germ plasm-specific miRNA *miR-202-5p*, which regulates PGC migration through targeting *cdc42se1* to protect *cdc42* expression. However, knockdown of *cdc42se1* could not significantly rescue PGC migration in maternal *miR-202* mutant (M*miR-202*) embryos, indicating that there are other target genes of *miR-202-5p* required for the regulation of PGC migration. Herein, we revealed the transcriptional profiles of wild type and M*miR-202* PGCs and obtained 129 differentially expressed genes (DEGs), of which 42 DEGs were enriched cell migration-related signaling pathways. From these DEGs, we identified two novel *miR-202-5p* target genes *prdm12b* and *rab10*. Furthermore, we found that disruption of *rab10* expression led to significantly migratory defects of PGC by overexpression of *rab10* siRNA, or WT, inactive as well as active forms of *rab10* mRNA, and WT *rab10* overexpression mediated migratory defects could be partially but significantly rescued by overexpression of *miR-202-5p*, demonstrating that *rab10* is an important factor involved *miR-202-5p* mediated regulation of PGC migration. Taken together, the present results provide significant information for understanding the molecular mechanism by which *miR-202-5p* regulates PGC migration in zebrafish.

## 1. Introduction

Primordial germ cells (PGCs), the progenitors of gametes, segregate at early stages of embryonic development [1,2]. Shortly after specification, PGCs become motile and migrate towards the genital ridge where PGCs with somatic gonadal precursors form the gonad and enter gametogenesis to produce sperms or eggs [3,4,5,6,7]. In lower animals such as fruit fly, frog and zebrafish, the specification of PGC is determined by maternal germ plasm, a phase-separated liquid structure containing many specific factors such as coding mRNAs and their proteins (*dnd*, *vasa* and *nanos*) [8,9,10], long non-coding RNAs (*pgc* and *xlsirt*) [11,12,13], piRNAs [14], as well as microRNAs (*miR-9c*, *miR-969* and *miR-202-5p*) [15,16,17]. These germ plasm components play critical roles in asymmetric assembly of germ plasm, transcriptional repression, germ cell survival and direction of germ cell migration [1,18,19,20].

Like *Dictyostelium discoideum* or *Entamoeba histolytica*, zebrafish PGC migration utilizes the term of bleb, which is driven by hydrostatic pressure and cytoplasmic flow to emerge cells detaching from the actin cortex and forming a round protrusion in the cell front [21]. PGC migration requires high contractility, flexible protrusion formation and reduced adhesion levels. Firstly, the onset of PGC migration coincides with a decreased level of E-cadherin controlled by the regulator of G-protein signaling 14a protein [22]. Then, the morphology and motility of PGCs is maintained by three molecular modules including myosin light chain kinase (Mlck), Zeb1 and A5b, through elevating the cell contractility, decreasing the level of E-cadherin and reducing the level of membrane cortex attachment, respectively [23]. The Cxcl12a chemokine gradient is critical for directing PGC migration towards the genital ridge [24]. During PGC migration, the cell front is established by high pH, which needs the function of ca15b and filopodia [25], and an elevation in Rac1 activity to enhance actin filament assembly [26]. In addition, the members of GTPases superfamily ROCK/Rac/Cdc42 participate in the regulation of membrane invaginations, which is critical for pseudopod extension of zebrafish PGC [27,28]. The Reduction in the levels of cdc42 expression or cdc42 activity leads to migratory defects in zebrafish PGC [15,27]. Therefore, the regulation of PGC migration is a complex biological progress.

MicroRNAs (miRNAs) are a kind of endogenous 18–25 nt non-coding RNAs, which pair with the mRNA of protein-coding genes to achieve post-transcriptional regulation in almost all biological processes including germ cell development [29,30,31,32]. *let-7*, the first identified miRNA in animals, is a critical factor in the determination of germ versus somatic cell fates in mammals [33,34,35,36]. *miR-430* plays an important role in the clearance of maternal mRNAs during maternal to zygotic transition [37]. The *miR-430* regulation of Cxcl12 chemokine signaling confers genetic robustness in directing PGC migration towards the genital ridge [38]. Another miRNA *miR-202-5p* has been reported as a gonad-specific miRNA in zebrafish [39], Atlantic salmon [40], and medaka [41]. In zebrafish, *miR-202-5p* is a component of maternal germ plasm [16]. Loss of *miR-202-5p* results in an elevation in the level of cdc42se1, which decreases the expression of cdc42, ultimately, leading to severe migratory defects in PGCs [15]. However, knockdown of *cdc42se1* can’t effectively rescue PGC migration in maternal *miR-202* mutant (M*miR-202*) embryos, suggesting that there might exist other *miR-202-5p* target genes contributing to the regulation of PGC migration through different signaling networks.

To further understand the molecular mechanism underlying *miR-202-5p* mediated regulation of PGC migration, we analyzed the transcriptional files of wild type (WT) and M*miR-202* PGCs by transcriptome sequencing. The transcriptome data revealed 129 differentially expressed genes (DEGs), which were widely distributed in cell migration related to signaling pathways such as extracellular matrix, cell polarity, cytoskeleton, cell adhesion, cell motility and small GTPase activity. From these DEGs, we identified two novel *miR-202-5p* target genes *prdm12b* and *rab10*. Furthermore, loss- and gain-of-function experiments indicated that the proper balance of *rab10* was critical for PGC migration.

## 2. Results

### 2.1. Transcriptional Profiles in WT and MmiR-202 PGCs

Previously, we have generated a maternal *miR-202-5p* mutant (M*miR-202*) zebrafish line [15]. In this study, the PGCs were visualized by injection of *gfp-nanos 3′utr* mRNA in 1-cell embryos, and the GFP-labelled PGCs of WT or M*miR-202* embryos were isolated by fluorescence-activated cell sorting (FACS) at 24 h post fertilization (hpf). The collected PGCs were divided into three groups of WT and M*miR-202* PGC groups (300 PGCs each group) to construct six sequencing libraries. By Illumina sequencing, we obtained 546 M clean reads with about 91 M clean reads in each library, 89.2% of which were mapped to the zebrafish genome and annotated as 30,878 transcripts (Table 1).

**Figure 1:**
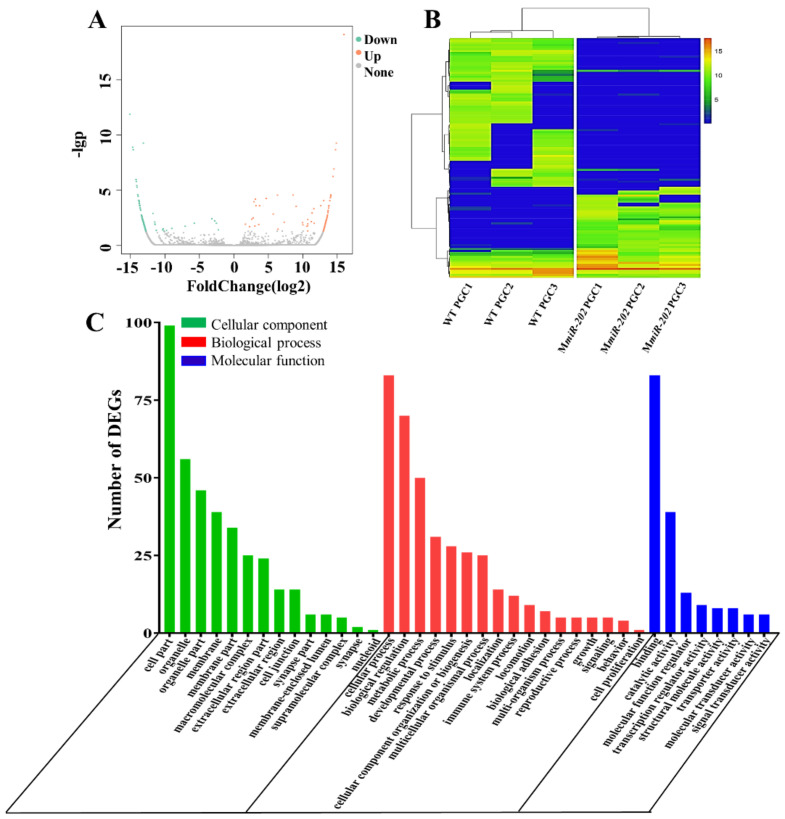
The expression pattern and GO annotation of DEGs. (**A**) Volcano plot of DEGs in WT and M*miR-202* PGCs. The up-regulated and down-regulated DEGs were respectively shown as red and green dots. (**B**) Heatmap for the cluster analysis of DEGs in WT and M*miR-202* PGCs. (**C**) The Gene Ontology annotation of all DEGs. The X-axis represents the 39 subcategories. The Y-axis shows the number of DEGs.

The expression level was calculated using FPKM (fragments per kilobase million mapped reads). To yield reliable results, only genes with FPKM ≥ 1 in at least three samples were subjected to analyze differentially expressed genes (DEGs). We obtained 129 DEGs including 46 up-regulated and 83 down-regulated DEGs in M*miR-202* PGCs compared to WT PGCs (Figure 1A,B). These DEGs were distributed in 39 GO terms and were mostly enriched in cell part, cellular process, biological regulation and binding (Figure 1C). In summary, we established the transcriptional files of WT and M*miR-202* PGCs.

### 2.2. Analysis of the DEGs Associated with PGC Migration

**Figure 2:**
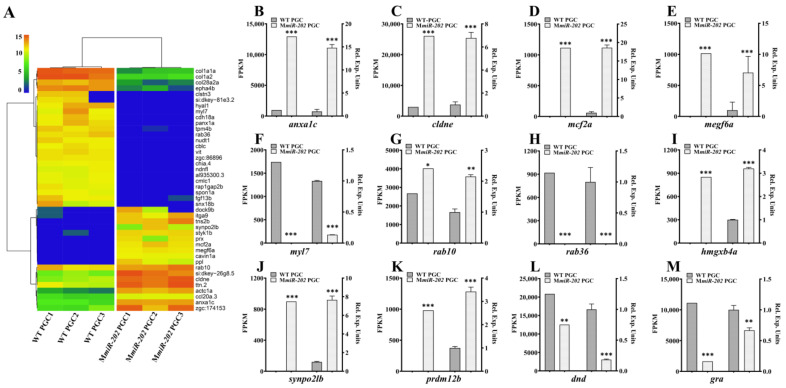
Heatmap of representative DEGs related to cell migration and verification of the expression patterns by qPCR analysis. (**A**) Two-dimensional hierarchical cluster of DEGs associated with cell migration. (**B**–**M**) Validation of the transcriptome by qRT-PCR analysis of representative genes including (**B**) *anxa1c*, (**C**) *cldne*, (**D**) *mcf2a*, (**E**) *megf6a*, (**F**) *myl7*, (**G**) *rab10*, (**H**) *rab36*, (**I**) *hmgxb4a*, (**J**) *synpo2lb*, (**K**) *prdm12b*, (**L**) *dnd* and (**M**) *gra*. The FPKM values (left) and the relative expression levels of qPCR (right) are shown. (**B**–**G**) The results were representative of more than three independent experiments in triplicate. Three reference genes *ef1a*, *β-actin* and *rpl13a* were used in every PCR examination to normalize gene expression levels with 2^-∆∆Ct^ method. The statistics were calculated and analyzed by Student’s *t*-test, * p < 0.05; ** p < 0.01; *** p < 0.001.

To explore genes involved in *miR-202-5p* mediated PGC migration, we performed bioinformatic analysis to predict functions of these DEGs and found that 42 DEGs were enriched in cell migration associated biological processes including extracellular matrix, cell polarity, cytoskeleton, cell adhesion, cell motility and small GTPase activity (Table 2, Figure 2A). Among these DEGs, eight DEGs were involved in the regulation of GTPase activity, a process essential for the regulation of PGC polarity [27]; seven DEGs were associated with the regulation of cadherin binding, and proper regulation of cadherin was required for the onset of PGC migration [42]. In addition, there were seven DEGs participating in assembly of myosin such as *myl7*, *tpm4b*, *rab36* and *cmlc1*, bleb formation *panx1a* as well as two positive regulator associated cell motility *cavin1a* and *rab36*, and the transcriptome data indicated that their expression levels were down-regulated in M*miR-202* PGCs compared to WT PGCs.

To validate the reliability of transcriptome data, we performed qPCR analysis of 12 representative DEGs including two PGC marker genes *dnd* and *gra*, four DEGs with over 10-fold change such as*hmgxb4a*, *prdm12b* and *myl7*, an ECM-related DEG *megf6a*, two cytoskeleton-related DEGs *anxa1c* and *synpo2lb*, three GTPase-related DEGs *rab10*, *mcf2a* and *rab36*, as well as cell adhesion-related DEG *cldne*. qPCR results exhibited a high consistency with the transcriptome data (Figure 2B–M): the transcriptional levels of *dnd*, *gra*, *myl7* and *rab36* were significantly downregulated, whereas *hmgxb4a*, *prdm12b*, *megf6a*, *rab10*, *mcf2a*, *anxa1c* and *synpo2lb* were significantly up-regulated in M*miR-202* PGCs compared to WT PGCs. Hence, the transcriptome data demonstrated that maternal loss of *miR-202-5p* led to dysregulation of multiple PGC migration-related signaling pathways.

### 2.3. rab10 and prdm12b Are Novel miR-202-5p Target Genes

**Figure 3:**
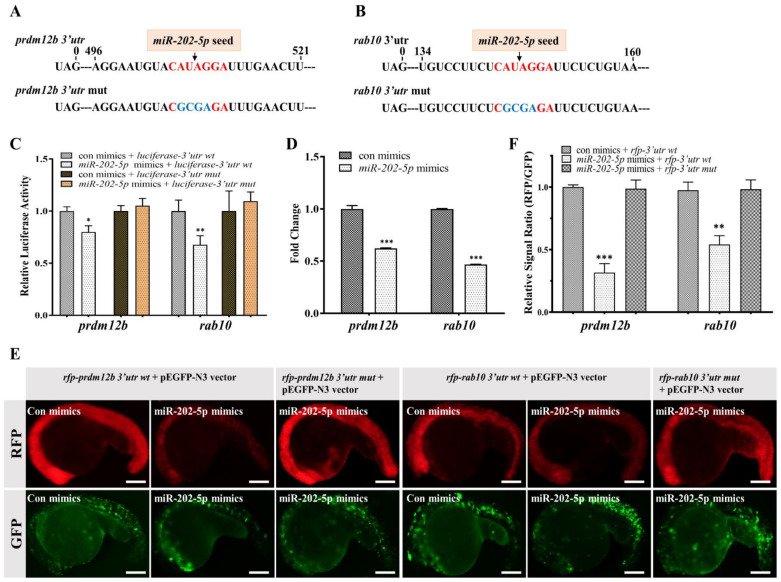
*miR-202-5p* directly regulates *prdm12b* and *rab10*. (**A**,**B**) The sequence information of the 3′utr of *prdm12b* and *rab10*. The canonical *miR-202-5p* binding sites and its mutant were shown in red and blue, respectively. (**C**) Relative luciferase activity in HEK293T cells transfected with psiCHECK2-*3′utr wt* (*prdm12b* or *rab10*) or psiCHECK2-*3′utr mut* (*prdm12b* or *rab10*) in the presence of control mimics (con mimics) or *miR-202-5p* mimics. (**D**) qRT-PCR analysis of *prdm12b* and *rab10* mRNA in 24hpf embryos injected with control or *miR-202-5p* mimics. (**E**) The representative images of embryos co-injected with mRNAs of *rfp-prdm12b 3′utr wt*, *rfp-prdm12b 3′utr mut*, *rfp-rab10 3′utr wt* or *rfp-rab10 3′utr mut* in the presence of control or *miR-202-5p* mimics. All embryos were co-injected with pEGFP-N3 vector as a control. (**F**) The relative signal ratio of RFP/GFP in the experiment presented in (**E**). (**C**,**D**,**F**) The results were representative of more than three independent experiments in triplicate. *β-actin* was used as an internal control to normalize gene expression levels with 2^-∆∆Ct^ method * p < 0.05; ** p < 0.01; *** p < 0.001.

To identify novel *miR-202-5p* target genes associated with PGC migration, we screened whether the 3′ untranslated region (UTR) of any DEG contained *miR-202-5p* binding sites by bioinformatic analysis. We found that the 3′UTR of *rab10* and *prdm12b* contained the canonical seed sequences of *miR-202-5p* (Figure 3A,B). Next, we employed luciferase and RNA reporter systems to clarify whether *rab10* and *prdm12b* are subjected to *miR-202-5p* regulation. Firstly, we constructed recombinant psiCheck2 plasmids by linking WT or mutant 3′UTR of *rab10* or *prdm12b* to the open reading frame (ORF) of luciferase and transiently transfected them to HEK293T cells. Dual luciferases assay indicated that the relative activity of luciferase fusing WT 3′UTR of *prdm12b* or *rab10* was significantly decreased in *miR-202-5p* mimics transfected HEK293T cells, whereas mutation of *miR-202-5p* binding sites significantly attenuated the repression (Figure 3C). Secondly, we employed an RNA reporter by linking the ORF of red fluorescent protein (RFP) with WT or mutant 3′UTR of *rab10* or *prdm12b* and co-injected it with *miR-202-5p* mimics or control mimics into 1-cell embryos. Quantitative pixel-intensity analysis revealed a significant decrease of RFP in the presence of *miR-202-5p* mimics, whereas the decrease was abolished by the mutation of *miR-202-5p* binding sites (Figure 3E,F). Thus, as previously shown for *cdc42se1* and *trim25* [15,43], the 3′UTRs of *rab10* or *prdm12b* were responsible for conferring negative regulation on the translation of linked ORFs through the action of *miR-202-5p*. Consistently, qPCR analysis of the RNA extracted from *miR-202-5p* or control mimic-injected embryos revealed a significant decrease of endogenous *rab10* or *prdm12b* in *miR-202-5p* mimic-injected embryos (Figure 3D), meanwhile there was a significant elevation of *rab10* or *prdm12b* in M*miR-202* PGCs (Figure 2G,K). These data indicated that *rab10* or *prdm12b* are *miR-202-5p* target genes.

### 2.4. The Balance of rab10 Is Critical of PGC Migration

**Figure 4:**
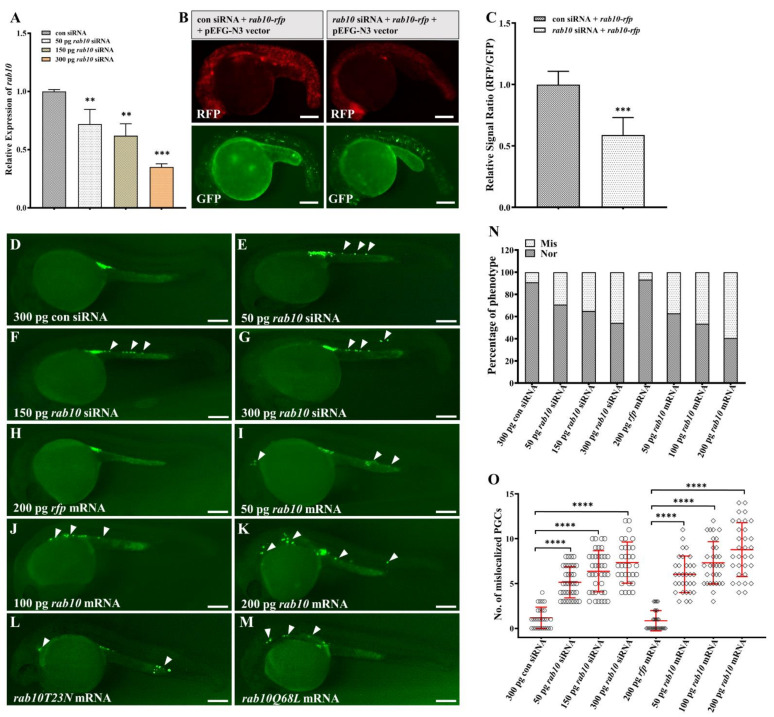
Disruption of *rab10* balance leads to PGC mislocalization. (**A**) qRT-PCR analysis of *rab10* mRNA in 24hpf embryos injected with control siRNA (con siRNA) or gradient *rab10* siRNA (50, 150, 300 pg). (**B**) Co-injection of *rab10* siRNA decreased expression of RFP in embryos injected with *rab10*-*rfp* mRNA compared to embryos co-injected with control siRNA. All embryos were co-injected with pEGFP-N3 vector as a control. (**C**) Quantitative representation of the normalized signal intensity in the experiment presented in (**B**). (**D**–**M**) Representative images of embryos injected with 300 pg con siRNA (**D**), 50 pg *rab10* siRNA (**E**), 150 pg *rab10* siRNA (**F**), 300 pg *rab10* siRNA (**G**), 200 pg *rfp* mRNA (**H**), 50 pg *rab10* mRNA (**I**), 100 pg *rab10* mRNA (**J**), 200 pg *rab10* mRNA (**K**), *rab10T23N* mRNA (**L**), and *rab10Q68L* mRNA (**M**). Arrowheads indicate mislocalized PGCs. (**N**) The percentage of PGC phenotypes in embryos with (**D**–**K**). (**O**) The number of mislocalized PGCs in each embryo with the experiment presented in (**D**–**K**). The average number of mislocalized PGCs were showed in red line by mean ± sd. (**A**,**C**,**N**,**O**) The results were representative of more than three independent experiments in triplicate. *β-actin* was used as an internal control to normalize gene expression levels with 2^−∆∆Ct^ method. ** *p* < 0.01; *** *p* < 0.001, **** *p* < 0.0001. Scale bar, 100 µm.

To investigate the possible roles of *rab10* and *prdm12b* in zebrafish PGC development, we employed loss- and gain-of-function by injection of corresponding siRNA and mRNA, respectively. Firstly, qPCR analysis on the extracted RNA from control or *rab10* siRNA injected embryos revealed a significant reduction in levels of *rab10* in *rab10* siRNA injected embryos, and the reduction relied on the dosages of *rab10* siRNA (Figure 4A). Secondly, we employed the fluorescence reporter of *rab10*-*rfp* mRNA and co-injected it with control or *rab10* siRNA into 1-cell embryos. Quantitative pixel-intensity analysis revealed a significant decrease in the levels of RFP in *rab10* siRNA injected embryos compared to that in control siRNA injected embryos (Figure 4B,C). These results demonstrated that *rab10* siRNA is efficient to decrease endogenous *rab10* expression in vivo.

Next, we investigated whether knockdown of *rab10* affected PGC migration in the PGC fluorescence labeling transgenic line *(kop*: *egfp-UTR-nos3*). We observed a significant mislocalization of PGCs in *rab10* siRNA injected embryos at 24 hpf (Figure 4D–G). We observed that the proportion of embryos with mislocalized PGCs and average number of mislocalized PGCs was 29.27% and 5.1, 34.88% and 6.4, 45.72% and 7.3 in 50, 150 and 300 pg *rab10* siRNA injected embryos, respectively (Figure 4N,O). Because the expression of *rab10* was elevated in M*miR-202* PGC, we speculated that overexpression of *rab10* also affected PGC migration. To verify it, ectopic overexpression of *rab10* was performed by injection of WT *rab10* mRNA. We observed that ectopic overexpression of *rab10* led to a significantly migratory defect of PGCs. The proportion of embryos with mislocalized PGCs and average number of mislocalized PGCs in 50, 100 and 200 pg *rab10* mRNA was 37.0% and 6.5, 46.4% and 7.5, 59.5% and 8.6, respectively (Figure 4H–K,N,O). Therefore, the migratory defect of PGC exhibited a significant dosage effect associated with the injected dosage of either *rab10* siRNA or mRNA. Rab proteins alternate between GTP- and GDP-bound states, which functions as the molecular switch in regulation of cell trafficking and movement [44]. It has been reported that canine rab10 mutants rab10T23N and rab10Q68L prefer to maintain inactive GDP- and active GTP-bound state, respectively [45]. Homological comparison indicated that the two functional sites were conserved in zebrafish rab10 proteins. We constructed inactive and active mRNA mutants of *rab10T23N* and *rab10Q68L* and then injected them in 1-cell embryos, respectively. We observed that injection of either *rab10T23N* or *rab10Q68L* mRNA resulted in migratory defects in PGCs (Figure 4L,M).

**Figure 5:**
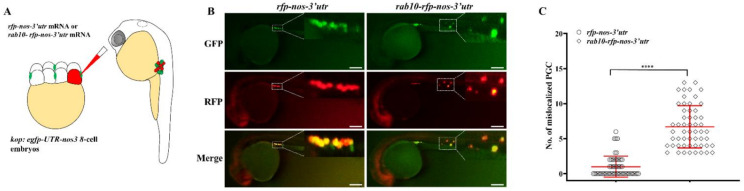
PGC-specific overexpression of *rab10* leads to PGC mislocalization. (**A**) *rab10-rfp-nos-3′utr* mRNA was injected into 8-cell embryos of *kop: egfp-UTR-nos3* lines. (**B**) Representative images of PGCs in embryos injected with *rfp-nos* mRNA or *rab10-rfp-nos-3′utr* at prim 5 stage. (**C**) The number of mislocalized PGCs in each embryo injected with *rfp-nos* mRNA or *rab10-rfp-nos-3′utr* at prim 5 stage. (**C**) The results were representative of more than three independent experiments in triplicate. The average number of mislocalized PGCs were showed in red line by mean *±* sd. **** *p* < 0.0001. Scale bar, 100 µm.

Furthermore, PGC-specific overexpression of *rab10* by injection of *rab10-rfp-utr-nos3* mRNA into 8-cell embryos led to a significant PGC mislocalization (Figure 5A–C). In summary, the proper balance of rab10 ensures PGC migration in zebrafish.

### 2.5. miR-202-5p Negatively Regulates rab10 in PGC Migration

**Figure 6:**
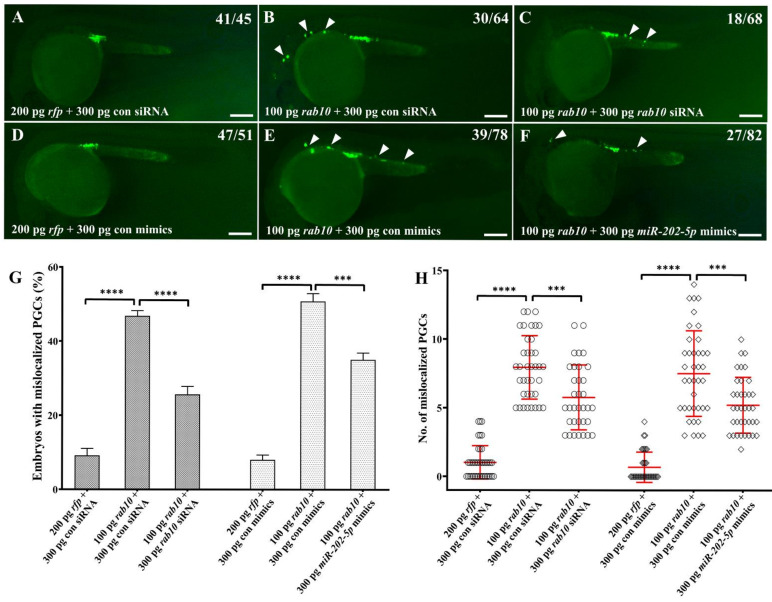
Overexpression of *miR-202-5p* partially rescues PGC migration in embryos overexpressing *rab10*. (**A**–**F**) Representative images of PGCs in embryos injected with 200 pg *rfp* mRNA and 300 pg control siRNA or control mimics (**A**,**D**), 100 pg *rab10* mRNA and 300 pg control siRNA or control mimics (**B**,**E**), 100 pg *rab10* mRNA and 300 pg *rab10* siRNA or *miR-202-5p* mimics (**C**,**F**). Arrowheads indicate mislocalized PGCs. The corresponding phenotypes in different groups of embryos were shown in the upper right of the panels (**A**–**F**). (**G**) The percentage of embryos with mislocalized PGCs in (**A**–**F**). (**H**) The number of mislocalized PGCs in each embryo in (**A**–**F**). (**G**,**H**) The results were representative of more than three independent experiments in triplicate. The average number of mislocalized PGCs were showed in red line by mean ± sd. *** *p* < 0.001, **** *p* < 0.0001. Scale bar, 100 µm.

To clarify the regulatory relationship between *miR-202-5p* and *rab10* during PGC migration, we injected *rab10* siRNA or *miR-202-5p* mimics to rescue the migratory defect of PGCs in embryos injected WT *rab10* mRNA. Compared to WT *rab10* mRNA injected embryos, co-injection of *rab10* mRNA with either *rab10* siRNA or *miR-202-5p* mimics could significantly reduce both the proportion of embryos with mislocalized PGCs and average number of mislocalized PGCs (Figure 6), although some embryos still had a modest number of mislocalized PGCs. Therefore, overexpression of *miR-202-5p* could partially rescue PGC migratory defects caused by overexpression of *rab10*.

### 2.6. Knockdown of rab10 Fails to Rescue PGC Migration in MmiR-202 Embryos

**Figure 7:**
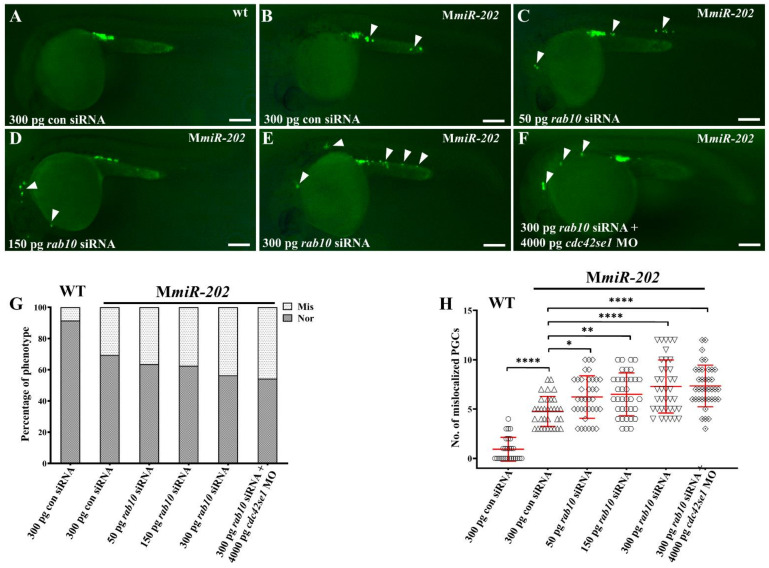
*rab10* siRNA and *cdc42se1* MO failed to rescue mislocalized PGCs in embryos caused by maternal absence of *miR-202-5p*. (**A**) Representative images of PGCs in WT embryos injected with 300 pg con siRNA; (**A**) Representative images of PGCs in Mmir-202 embryos injected with 300 pg con siRNA (**B**), 50 pg *rab10* siRNA (**C**), 150 pg *rab10* siRNA (**D**), 300 pg *rab10* siRNA (**E**), and 300 pg *rab10* siRNA + 4000 pg *cdc42se1* MO (**E**). Arrowheads indicate mislocalized PGCs. (**G**) The percentage of embryos with mislocalized PGCs with (**A**–**F**). The genotypes of the embryos were shown upper right in each panel. (**H**) The number of mislocalized PGCs in each embryo in the experiment presented in (**A**–**F**). (**G**,**H**) The results were representative of more than three independent experiments in triplicate. The average number of mislocalized PGCs were showed in red line by mean ± sd. * *p* < 0.05, ** *p* < 0.01, **** *p* < 0.0001. Scale bar, 100 µm.

On the other hand, we investigated whether knockdown of *rab10* could rescue the migratory defect in M*miR-202* PGC. Different dosages (50, 150 and 300 pg) of *rab10* siRNA were injected into 1-cell M*miR-202* embryos. In 300 pg control siRNA injected WT embryos, there were 6.21% embryos with an average number (0.91) of mislocalized PGC (Figure 7A). On the other hand, the proportion of embryos with mislocalized PGCs and average number of mislocalized PGCs was 30.56% and 4.76 in control siRNA injected M*miR-202* embryos (Figure 7B). However, the proportion of embryos with mislocalized PGCs and average number of mislocalized PGCs increased to 36.36% and 6.22, 37.50% and 6.50, 43.59% and 7.29 in 50, 150 and 300 pg *rab10* siRNA injected M*miR-202* embryos, respectively (Figure 7C–E,G,H). Therefore, knockdown of *rab10* in M*miR-202* embryos resulted in a severer defect in PGC migration compared to M*miR-202* embryos. Previously, we have found that *miR-202-5p* protects PGC migration by targeting and repressing the expression of *cdc42se1* [15]. Therefore, we attempted to rescue PGC migration in M*miR-202* embryos by double knockdown of *rab10* and *cdc42se1*. However, we observed that the proportion of embryos with mislocalized PGCs and average number of mislocalized PGCs further increased to 45.71% and 7.34 in 300 pg *rab10* siRNA and 4000 pg *cdc42se1* MO co-injected M*miR-202* embryos, demonstrating that double knockdown of *rab10* and *cdc42se1* led to a stronger migratory defect in the PGC migration of M*miR-202* embryos (Figure 7F–H). Therefore, the migratory defect caused by maternal loss of *miR-202-5p* was complex.

## 3. Discussion

*miR-202-5p* is a conserved in animals, which plays an important role in reproduction [41], gametogenesis [46,47], immunology [43], tumorigenesis [48,49], and cell migration [15,50,51]. In mammals, *miR-202* is specific to Sertoli cell and might play an important role in the interaction between germ cell and Sertoli cell during spermatogenesis [46,52]. In frog, *miR-202-5p* is a germline-enriched miRNA in early stage of oogenesis [53]. Chicken *miR-202-5p* is highly expressed in spermatogonia rather than PGCs and might regulate germ cell development by targeting LIMK2 [47]. The expression patterns of *miR-202-5p* are divergent among fish species. In Japanese medaka (*Oryzias latipes*), *miR-202-5p* is co-localized with *sox9* in primordial follicles. Loss of *miR-202* impairs early folliculogenesis, decreases the number of mature follicles and leads to a dramatic reduction in female fecundity [41]. In zebrafish, *miR-202-5p* is a component of germ plasm. Loss of *miR-202* seems to not affect zebrafish fertility but results in a significant defect in PGC migration [16]. Previously, we have revealed a mechanism by which *miR-202-5p* uses to regulate PGC migration. *miR-202-5p* negatively regulates *cdc42se1* to ensure the proper expression of cdc42 in PGCs. However, it remains vague whether there are other mechanisms for *miR-202-5p* regulating PGC migration by other target genes. In this study, we performed transcriptome sequencing of WT and M*miR-202* PGCs and obtained 146 DEGs. Furthermore, we found that *rab10* acts as a downstream target gene of *miR-202-5p* and plays an important role in PGC migration. Therefore, we revealed a novel mechanism underlying *miR-202-5p* regulation of PGC migration.

Zebrafish PGC forms at about 4 hpf, initiates migration at 4.5 hpf and reaches the genital ridge at 24 hpf [54,55]. It is better to collect PGCs during the whole migratory process (4−24 hpf) to analyze the dynamic gene expression change in WT and M*miR-202* PGCs. However, because of lacking the PGC-specific transgenic *miR-202*^−/−^ line, it is difficult to collect M*miR-202* PGCs from the onset of PGC migration. The 3′UTRs of *nos1*, *dnd* and *tdrd7* regulate differential localization between soma and PGCs [8,9,56]. However, in *gfp-nanos 3′utr* mRNA injected embryos, the PGC-specific fluorescence could be found at the late segmentation period, usually at 24 hpf. Therefore, we collected PGCs in *gfp-nanos-3′utr* mRNA injected WT and M*miR-202* embryos at 24 hpf to ensure the purity of isolated PGCs. The transcriptome results revealed that 42 DEGs in WT and M*miR-202* PGCs were significantly enriched in cell migration-associated biological processes (Table 2). The motility of PGC requires a decrease of cadherin-mediated tight junction and a proper regulation of myosin-based contractility [3,23,26]. However, we found a remarkable elevation in the levels of tight junction-associated genes including *cldne*, *itga9* and *styk1b*, but a reduction in the levels of myosin, polarity and bleb associated genes including *myl7*, *tpm4b*, *rab36*, *cmlc1*, *panx1a*, *cavin1a*, *fgf13b* and *rab36* in M*miR-202* PGCs compared to WT PGCs. Chemokine-guided signaling pathway is essential for the direction of PGC migrating towards genital ridges [24,38]. We found two chemokine-associated genes *chia.4* and *ccl20a.3* were differentially expressed in WT and M*miR-202* PGCs, suggesting that maternal loss of *miR-202* might disrupt chemokine signaling to affect PGC migration. In addition, many DEGs were enriched in the regulation of cell adhesion, cytoskeleton, ECM and GTPase activity. Together, the transcriptome files of WT and M*miR-202* PGCs provided global and useful data for understanding the potential signaling pathways involved in *miR-202-5p* regulation of PGC migration.

Because miRNAs rarely encode functional protein products, they usually initiate hierarchical biological events by binding to the mRNAs of target genes to direct post-transcriptional repression. Therefore, identifying target genes will be helpful to understand how *miR-202-5p* regulates PGC migration. From these DEGs, we identified two novel *miR-202-5p* target genes *prdm12* and *rab10*. The members of PR domain-containing (PRDM) family are critical in regulation of DNA methylation and stem cell population maintenance. PRDM1 and PRDM14 are critical determinants of mammalian germ cell lineages from embryonic cells [57,58]. However, zebrafish PRDM family mainly regulate craniofacial development [59]. We found that overexpression or knockdown of *prdm12b* had no significant effect on PGC development in zebrafish (data not shown), suggesting PRDM family exert divergent roles in mammals and fish.

The members of small GTPase superfamily participate in almost every aspect of cell biology, which is comprised of five families conserved across eukaryotes: Ras, Rho, Rab, Arf, and Ran [60]. They alternate between GTP- and GDP-bound states, which act as molecular switches in regulation of polarity, cytoskeleton change, membrane invaginations in all eukaryotic cells [44,60]. The activity of RhoA/ROCK is essential for correct localization of germ plasm mRNAs at cleavage stage [28]. The Rac1 activation is essential for establishing the cell front and polarity of PGCs [61]. Cdc42 and ROCK regulate membrane invaginations, which is essential for motility of PGCs [62]. Inhibition of Rac/ROCK/Cdc42 activities by expression domain negative proteins significantly impairs PGC motility and leads to ectopic localization of PGCs. Rab proteins are the largest branch of Ras superfamily, which is critical in the regulation of vesicle formation and trafficking [44]. The Clathrin- and Rab5-mediated endocytosis is required for activation of Rac, and the Rab5-to-Rac circuitry controls the morphology of motile PGCs in zebrafish [63]. Other members of Rab family participate in regulation of gametogenesis. Rab7 plays an important role in actin dynamics and mitochondria function in oocyte meiosis [64]. *rab10* binds with the male germ cell-specific Rab GTPase-activating protein (MGCRABGAP) and the MGCRABGAP-RAB complex localizes in the manchette structure of mature sperm, indicating a possible role in spermatogenesis [65]. However, whether *rab10* participates in PGC development remains unclear. Herein, we found that disruption of *rab10* balance through siRNA mediated reduction, overexpression of WT *rab10*, inactive or active *rab10* mutants led to PGC mislocalization, demonstrating that proper expression of *rab10*, especially for the balance of *rab10* GDP- and GTP-bound states, is critical for PGC migration (Figure 4). Because *rab10* is negatively regulated by *miR-202-5p*, we used *miR-202-5p* mimics to rescue PGC migratory defects in *rab10* overexpressed embryos. However, overexpression of *miR-202-5p* partially rescued the migratory defects caused by *rab10* overexpression (Figure 6), which might be because although *miR-202-5p* might rescue the expression level of *rab10*, the balance between rab10 GDP- and GTP-bound states remains in disorder. Taken together, *miR-202-5p* mediated balance of rab10 is important for PGC migration.

Finally, we observed that knockdown of *rab10* could not significantly rescue PGC migration and even led to a severer migratory defect of PGCs in M*miR-202* embryos, no matter knockdown of *cdc42se1* or not. There might be two possible explanations for why we failed to rescue PGC migration by knockdown of *rab10* and *cdc42se1* in M*miR-202* embryos. Firstly, injection of *rab10* siRNA could rescue the expression level of *rab10* but might cause a more significant disorder in the balance between rab10 GDP- and GTP- bound states in M*miR-202* embryos. Secondly, loss of *miR-202-5p* led to a complex dysregulation of downstream signaling networks besides *rab10* and *cdc42se1*, such as the chemokine signaling. Chemokine-guided cell migration is essential for directing PGC migration towards the genital ridge [38]. Herein, we found that the *ccl20a.3* chemokine was differentially expressed between WT and M*miR-202* PGCs, providing possible evidence suggesting that *miR-202-5p* might regulate PGC migratory behavior via chemokine signaling. However, which type of cell expressing *ccl20a.3* chemokine receptor–ccr6 during early embryogenesis; whether and how ccr6 positive cells contribute to PGC migration towards the genital ridge needs to be further investigated. In summary, the present work provided transcriptional profiles of M*miR-202* PGCs, revealed the differentially expressed signaling pathways between WT and M*miR-202* PGCs, and identified two novel *miR-202-5p* target genes *rab10* and *prdm12b*. Loss- and gain-of-function demonstrated that *rab10* is required for PGC migration. The present work provides important data for understanding the molecular mechanism underlying *miR-202-5p* regulation of zebrafish PGC.

## 4. Materials and Methods

### 4.1. Ethics Statement

All procedures with zebrafish were approved by the Ethics Committee of Sun Yat-sen University (31771587, 20 February 2017), and the methods were carried out in accordance with the approved guidelines.

### 4.2. Zebrafish Strains and Cell Lines 

The AB line and transgenic line *Tg kop*: *egfp-UTR-nos3* zebrafish were purchased from the China Zebrafish Resource Center. The maternal *miR-202* mutant (M*miR-202*) zebrafish has been generated in our previous study [15]. Fish were raised at 28 °C with 10 h (h) darkness and 14 h light in the zebrafish circulatory breeding system.

HEK 293T cells were cultured at 37^°^C in Dulbecco’s modified Eagle’s medium (DMEM) supplied with 10% FBS (Invitrogen, Carlsbad, CA, USA) under a humidified atmosphere of air containing 5% CO_2_.

### 4.3. Isolation of PGC and Transcriptome Analysis

To label the PGCs with green fluorescence protein (GFP), 400pg *gfp-nos 3′utr* mRNA was injected into 1-cell WT and M*miR-202* embryos. At 24 hpf, the embryos were digested to single-cell suspension by trypsin according to the zebrafish book (http://zfin.org/zf_info/zfbook/zfbk.html, 4th version, accessed date: 7 October 2021). A total of 900 WT and 900 M*miR-202* PGCs were collected by FACS based on the GFP signal. The isolated PGCs were equally divided into three technical replicates of WT PGCs and M*miR-202* PGCs groups for Illumina sequencing on the MGIseq2000 platform (Anoroad genome, Beijing, China).

### 4.4. Transcriptome Sequencing and Bioinformatic Analysis

After sequencing, clean reads were obtained with Perl scripts to clear raw read containing more than 5 adapter-polluted bases, the number of N bases accounting for more than 5%, as well as the phred quality value less than 19 accounting for more than 15%. Clean reads were mapped to the reference genome and annotation file downloaded from Ensembl (*Danio rerio* GRCz10.89.gtf) with HISAT2 [66]. Read Count for each gene in each sample is counted by HTSeq and gene expression level was calculated using FPKM (Fragments Per Kilobase Millon Mapped Reads). To yield reliable results, genes with FPKM ≥ 1 in at least three samples were taken for differential expression analysis between WT and M*miR-202* PGCs with DEGseq with BH method (false discovery rate correction with Benjamini–Hochberg), and genes with q ≤ 0.05 and |log2_ratio| ≥ 1 were identified as differentially expressed genes. The biological signaling pathway of DEGs was analyzed by functional enrichment in GO terms (Gene Ontology, http://geneontology.org/, 1 January 2021) and KEGG pathways (Kyoto Encyclopedia of Genes and Genomes, http://www.kegg.jp/, Release 97.0, 1 January 2021). GO terms with q < 0.05 were considered significantly enriched.

### 4.5. Embryo Collection and RNA Isolation

For RNA isolation, WT embryos, embryos injected control or *miR-202-5p* mimics, control siRNA or different dosages (50/150/300 pg each embryo) of *rab10* siRNAs, as well as embryos injected with *gfp* mRNA or different dosages (50/100/200 pg each embryo) of *rab10* mRNA were collected at 24 hpf, frozen in liquid nitrogen and stored in −80 °C. The collected embryos were homogenized in 1 mL of trizol reagent (Invitrogen, Carlsbad, CA, USA), and total RNA was extracted according to the manufacturer’s instructions.

### 4.6. Reverse Transcription and qPCR Analysis

To examine the expression levels of predicted DEGs between WT and M*miR-202* PGCs, the RNA of collected PGCs was reverse-transcribed with single-cell sequence-specific amplification kit to establish first the cDNA library (Vazyme, Nanjing, China). The extracted RNA of other embryos was reverse-transcribed into single-strand cDNA with primescript™ first-strand cDNA synthesis kit (Takara, Kyoto, Japan). To avoid the error caused by single reference genes in single-cell PCR validation, we used three common reference genes including *ef1a*, *β-actin* and *rpl13a* to normalize gene expression levels using 2^−∆∆Ct^ method in every PCR examination. The results were representative of more than three independent experiments in triplicate. Each independent experiment was performed in triplicate.

qPCR analysis was performed on the LightCycler480 II (Roche, Basel, Switzerland) with the chamQ universal SYBR qPCR master mix (Vazyme, Nanjing, China) as previously described [67]. Primer sequences were listed in Table A1.

### 4.7. Plasmid Construction and mRNA Transcription

For luciferase or RFP reporters, the 3′UTRs of *rab10* (ENSDARG00000046106) or *prdm12b* (ENSDARG00000007430) were cloned and fused to the ORF of luciferase and RFP in the psiCHECK2 and pCS2 plus (pCS2+) vectors, respectively.

For universal *rab10* overexpression, full ORF and 3′UTR of *rab10* was inserted to pCS2+ vector. To specifically overexpress *rab10* in PGCs, the ORF of *rab10* and *gfp* as well as the 3′UTR of *nanos3* were inserted to pCS2+ vector to generate the *pCS2-rab10-gfp-nos 3′utr* plasmid.

All the point mutants including *miR-202-5p* binding sites within the 3′UTRs of *rab10* or *prdm12b*, and the inactive or active *rab10* mutants were constructed with hieff mut^tm^ multi-site-directed mutagenesis kit (Yeasen, Shanghai, China) as previously described [15,45].

For mRNA synthesis, recombinant pCS2+ plasmids were linearized with NotI and transcribed using SP6 mMESSAGE mMACHINE Kit (Invitrogen, Carlsbad, CA, USA) according to the manufacturer’s instructions.

### 4.8. Micro-Injection

To knockdown *rab10*, three dosages (50/150/300 pg each embryo) of *rab10* siRNA (GCCAACATCAACATCGAGA) or 200 pg control siRNA (RiboBio, Guangzhou, China) was respectively injected into 1-cell WT embryos to knockdown endogenous *rab10*, and the embryos were collected for qPCR analysis of *rab10* expression. Subsequently, the *rab10* siRNA was injected into 1-cell embryos of *kop*: *egfp-UTR-nos3* line.

To overexpress *rab10*, different dosages (50/100/200 pg each embryo) of *rab10*, *rab10T23N* and *rab10Q68L* mRNA were respectively injected into 1-cell embryos of *Tg kop*: *egfp-UTR-nos3* line.

To rescue PGC migration in embryos injected *rab10* mRNA or *rab10* siRNA, 50 pg *rab10* siRNA or 100 pg *rab10* mRNA were co-injected into 1-cell embryos of *Tg kop*: *egfp-UTR-nos3* line, and PGC migration was observed at 24 hpf. Furthermore, 300 pg *miR-202-5p* mimics were co-injected to rescue PGC migration in *rab10* mRNA injected embryos.

To rescue migratory defects in M*miR-202* PGCs, embryos were injected with different dosages (50/150/300 pg each embryo) of *rab10* siRNA or 4000 pg *cdc42se1* morpholino, respectively.

To confirm *rab10* or *prdm12b* were *miR-202-5p* target genes, 200 pg *rfp-prdm12b-3′utr-wt*, *rfp-prdm12b-3′utr-mut, rfp-rab10-3′utr-wt* or *rfp-rab10-3′utr-mut* mRNA was co-injected with control or *miR-202-5p* mimics. At 24 hpf, representative images of corresponding embryos were captured with the stereo fluorescence microscope (Nikon, SMZ800N, Japan). And the average signal intensity of RFP was measured by Image J (version 1.51).

All the PGC migration was examined at 24 hpf with the stereo fluorescence microscope (SMZ800N, Nikon, Kyoto, Japan). All experiments were performed in more than three independent experiments in triplicate. Each independent experiment was performed in triplicate.

### 4.9. Dual Luciferase Reporter Assay

At 70–80% confluence, HEK293T cells seeded in 24-well plates were transiently co-transfected 10 ng *psiCHECK2-3’UTR-prdm12b-WT* (*psiCHECK2-3’UTR-rab10-WT*) or *psiCHECK2-3’UTR-prdm12b-seed-Mut* (*psiCHECK2-3’UTR-rab10-seed-Mut*) plasmids and 100nM control or *miR-202-5p* mimics with Lipofectamine 8000 (Beyotime, Shanghai, China). The cells were lysed 24 h post-transfection, and the luciferase activity was detected by the dual-luciferase reporter analysis system (Promega, Madison, WI, USA). The results were representative of more than three independent experiments, each performed in triplicate. Each independent experiment was performed in triplicate.

### 4.10. PGC Phenotype Observation

At 24 hpf, PGCs of different groups of embryos were observed with the stereoscopic fluorescence microscope. The delineation of normal and mislocalized phenotype were distinguished as following: embryos with less than 3 ectopic PGCs were regarded as normal, whereas embryos with more than 3 ectopic PGCs were regarded as mislocalization. At least four repeated groups of embryos (30–50 embryos in each group) were observed.

### 4.11. Statistics Analysis

The statistics were calculated and analyzed with SPSS version 20. The qPCR results were analyzed by Student’s *t*-test, and the phenotypes in different experimental groups were analyzed by one-way ANOVA. *p* < 0.5 was considered a statistically significant difference.

## Figures and Tables

**Table 1:** The quality information of transcriptome sequencing.

Sample	Raw Reads	Clean Reads	Clean Reads Rate (%)	Q30 (%)	Mapping Rate (%)
WT PGC1	92,981,332	90,378,978	97.20	92.49	89.66
WT PGC2	94,130,668	91,742,980	97.46	92.99	90.48
WT PGC3	93,087,162	91,203,598	97.98	93.34	88.52
M*miR-202* PGC1	94,567,190	91,905,698	97.19	93.48	88.70
M*miR-202* PGC2	95,092,164	91,571,918	96.30	93.25	88.75
M*miR-202* PGC3	95,230,438	92,724,062	97.37	93.26	88.91

**Table 2:** The DEGs associated with GO terms of migratory events.

GO Terms Related to Migratory Events	DEGs
Bleb	*panx1a*
Cell polarity	*fgf13b*
Cell motility	*rab36*, *cavin1a*
Chemokine	*chia.4*, *ccl20a.3*
Cadherin	*cdh18a*, *clstn3*, *cblc*, *si:dkey-81e3.2*, *ppl*, *prx*, *dock9b*
Cell adhesion	*cldne*, *col28a2a*, *cdh18a*, *clstn3*, *hyal1*, *spon1a*, *si:dkey-81e3.2*, *itga9*, *styk1b*
Cytoskeleton	*al935300.3*, *actc1a*, *anxa1c*, *epha4b*, *mcf2a*, *ppl*, *prx*, *ttn.2*, *tpm4b*, *synpo2lb*, *si:dkey-81e3.2*, *si:dkey-26g8.5*, *zgc:86896*, *zgc:174153*
Extracellular matrix	*col1a1a*, *col1a2*, *col28a2a*, *fgf13b*, *vit*, *ndnfl*, *tns2b*, *itga9*, *si:dkey-81e3.2*, *megf6a*
GTPase activity	*dock9b*, *epha4b*, *mcf2a*, *nudt1*, *rap1gap2b*, *rab10*, *rab36*, *snx18b*
Myosin	*myl7*, *rab36*, *cmlc1*, *tpm4b*

## Data Availability

All the Illumina sequencing reads (SRA: PRJNA768286) have been deposited in the NCBI.

## Abbreviations

3′UTR	3′untranslated region
DEGs	differentially expressed genes
DMEM	Dulbecco’s modified Eagle’s medium
ECM	extracellular matrix
FACS	fluorescence activated cell sorting
FPKM	Fragments Per Kilobase Millon Mapped Reads
GFP	green fluorescence protein
GO	Gene Ontology
KEGG	Kyoto Encyclopedia of Genes and Genomes
miRNAs	MicroRNAs
Mlck	myosin light chain kinase
M*miR-202*	maternal miR-202 mutant
ORF	open reading frame
PGCs	Primordial germ cells
PRDM	PR domain containing
qPCR	Quantitative polymerase chain reaction
RFP	red fluorescent protein
WT	wild type

## Appendix A

**Table A1:** Sequences of primer used in this paper.

Description	Primer Name	Primer Sequence (5′-3′)
psiCHECK2	*rab10*-3′UTR-XhoI-F	CCGCTCGAGCACACACGCACACACAATCTGTTCTACT
	*rab10*-3′UTR-NotI-R	ATAAGAATGCGGCCGCCGTGAGTGTGAGAAACGTTAGAGATG
	*rab10*-3′UTR-seed Mut-R	GTTAGAGATGTGTTTTACAGAGAATCTCGCGAGAAGG
	*prdm12b*-3′UTR-XhoI-F	CCGCTCGAG ACCACACCACGTTATTTGCT
	*prdm12b*-3′UTR-NotI-R	ATAAGAATGCGGCCGCAGGTGAGCTAAATTGGCCG
	*prdm12b*-3′UTR-seed Mut-F	GGAATGTACGCGAGATTTGAACTTTTACG
	*prdm12b*-3′UTR-seed Mut-R	GTTCAAATCTCGCGTACATTCCTTTAC
pCS2 + mRNA synthesis	*rab10*-Bamh I-F	ACGGGATCCAGCTCAGATGGCGAAGAAGACCTATGATC
	*rab10*-Age I-R	ACTACCGGTGGACTGCAGCACTTGGTCTTCCAGCCT
	*rab10*-XhoI-R	CCGCTCGAGCGTGAGTGTGAGAAACGTTAGAGATG
	*rab10T23N*-F	TGATTGGCGATTCTGGGGTGGGAAAGAACTGCGTGCTGTT
	*rab10T23N*-R	TCATCGGAGAATCGGAACAGCACGCAGTTCTTTCCCACCC
	*rab10Q68L*-F	GCTACAGATATGGGATACAGCGGGGCTGGAACGGTTTCAC
	*rab10Q68L*-R	AGGTAGTGATGGTGTGAAACCGTTCCAGCCCCGCTGTATC
qRT-PCR	*ef1a*-F	GGCTGACTGTGCTGTGCTGATTG
	*ef1a*-R	CTTGTCGGTGGGACGGCTAGG
	*actin*-F	CGAGCTGTCTTCCCATCCA
	*actin*-R	TCACCAACGTAGCTGTCTTTCTG
	*rpl13a*-F	TCTGGAGGACTGTAAGAGGTATGC
	*rpl13a*-R	AGACGCACAATCTTGAGAGCAG
	*rab10*-RT-F	CGCCTTCAACACCACCTTTAT
	*rab10*-RT-R	GCATCCTCTCCACATCCTCAT
	*prdm12b*-RT-F	TCGCAGCTGGATGACTTACA
	*prdm12b*-RT-R	TCCCATACCACACAAGCAGT
	*anxa1c*-RT-F	ACACCTTGAAGACTGCCTGA
	*anxa1c*-RT-R	CCGAACGGCTCACAATGATT
	*cldne*-RT-F	GGGAAATGCACCAACTTCGT
	*cldne*-RT-R	CTCTGATGATGGTGTTGGCG
	*mcf2a*-RT-R	GCAGCGCTATTGAGTTCCTC
	*mcf2a*-RT-F	AGTCCTGTGTGATGTCCCAG
	*megf6a*-RT-F	CAGGGCAGGTTACAGACTCA
	*megf6a*-RT-R	GTCTGTCCTCACCAAGTCGA
	*myl7*-RT-F	TGCACAACTAGGGAAGCTGA
	*myl7*-RT-R	GGTCTGTGCCATTGAGCTTC
	*rab36*-RT-F	GCCGTAAAGATTGCAGCAGA
	*rab36*-RT-R	ACTGCCATCTCCGATCTGAG
	*hmgxb4a*-RT-F	TCGTCTCTGGGCATGTCTTT
	*hmgxb4a*-RT-R	CTGTCTCCTGAAGTCGGTGT
	*synpo21b*-RT-F	CAACCTACTCCAGCACCTCA
	*synpo21b*-RT-R	TGTGGAGGACTGAATGGCAT
	*dnd*-RT-F	GGCTAAGAAAGTGCTCGTGG
	*dnd*-RT-R	GCTGGGACGTCATAATGCAG
	*gra*-RT-F	CCTTAAAAGCACCGAGACCC
	*gra*-RT-R	AAGTATCTGGGCAGGTCACT
